# The interaction between CTGF and VEGF-A in the progression of intervertebral disc fibrosis

**DOI:** 10.4314/ahs.v24i4.36

**Published:** 2024-12

**Authors:** Wangbing Xu, Jiqin Zhong, Jianrong Jian, Faming Zhong

**Affiliations:** 1 Spinal surgery, Affiliated Hospital of Jiangxi University of Traditional Chinese Medicine, Nanchang, China; 2 Public Health Branch, Affiliated Hospital of Jiangxi University of Traditional Chinese Medicine, Nanchang, China; 3 Recovery physical therapy branch, Affiliated Hospital of Jiangxi University of Traditional Chinese Medicine, Nanchang, China

**Keywords:** Connective tissue growth factor, vascular endothelial growth factor -A, intervertebral disc, nucleus pulposus, fibrosis

## Abstract

**Background:**

Fibrosis in the extracellular matrix of nucleus pulposus (NP) is associated with intervertebral disc degeneration (IVDD). Both connective tissue growth factor (CTGF) and vascular endothelial growth factor (VEGF)-A are responsible for the pathological basis of NP fibrosis. Our study aims to verify the interaction between CTGF and VEGF-A in a vitro NP cell model.

**Methodology:**

Collected human NP tissues of different degeneration degree and isolated the NP cells from the non-degenerated NP tissues. Analysed the CTGF and VEGF-A gene expression in the naturally degenerated NP and IL-1β-induced degenerated NP cells. Additionally, interfered wit the CTGF and VEGF-A expression by exogenic protein treatment, siRNA transfection, or specific inhibitor. The expression of CTGF, VEGF-A, collagen I/II/III and aggrecan with protein or mRNA level was determined by immunological staining, western blotting and RT-PCR.

**Results:**

CTGF and VEGF-A highly expressed in the late-term of degeneration compared to the middle-term, and their expressions were synergistic. Upregulating one of CTGF and VEGF-A could induce the overexpression of the other one and collagen I/III, but suppressed collagen II and aggrecan expression; Besides, the suppression of one of them could inhibited another and collagen I/III expression.

**Conclusions:**

CTGF and VEGF-A increase in late IVDD. Prevent NP fibrosis by suppressing their interaction.

## Introduction

Intervertebral disc degeneration (IVDD) is a multi-factor-mediated age-related disease that is characterized by changes in the structure and function of the disc^1^. With the development of the degenerative process, it eventually leads to spinal diseases such as disc herniation, spinal canal stenosis, and spinal spondylolisthesis, causing acute or chronic low back pain^2^. Decreased nucleus pulposus (NP) cell activity, changes in extracellular matrix (ECM) components, and fibrous hyperplasia are all factors that lead to changes in the biological activity of the disc^3^. NP cells are differentiated from mesenchymal stem cells (MSCs) and have the role of maintaining the healthy metabolism of the NP, generating and balancing the ECM. Meantime, the NP cells also absorb nutrition and keep their normal functions through stable ECM^4^. The ECM is composed of a large number of water molecules, collagen, proteoglycans, and other small biological molecules. Nowadays, lots of evidence indicate fibrosis exists in the majority of degenerated discs^5^, ^6^. Fibrosis progression can dehydrate the NP, reduce stress compliance, and change the elastic and viscous properties of the disc^7^. The primary characteristic of NP fibrosis refers to the disorder of collagen meshwork by a decreased collagen II proportion and an upregulated percentage of collagen I and III.

The development of molecular biology technology has gradually revealed the molecular pathological mechanism of IVDD, including various cytokines that regulate NP cell growth and differentiation. In 1991, Bradham et al^8^ cloned connective tissue growth factor (CTGF/CCN2) from human umbilical vein endothelial cells under the induction of platelet-derived growth factor, which can stimulate fibroblast proliferation and promote collagen precipitation, and is widely present in many tissues and organs of the human body. Recent studies have shown that CTGF profoundly expresses in highly degenerated disc^5^, ^9^, which not only participates in the process of disc fibrosis but also shows its effect in promoting angiogenesis^10^. The expression of CTGF in various tissues is low under physiological conditions. But under pathological conditions, the level of CTGF can be significantly increased, especially under the stimulation of high mechanical stress^11^, ^12^.

During the fibrosis of the human intervertebral disc, new blood vessels grow, and CTGF promotes the formation of capillaries. Vascular endothelial growth factor (VEGF) is the central regulator of angiogenesis and plays a vital role in physiological and pathological angiogenesis. VEGF has been widely reported to be involved in the pathogenesis of IVDD, and the high expression of VEGF in the disc marks the beginning of IVDD. Due to the complex role, there is no unified view of the effects of GTGF and VEGF in IVDD. On the contrary to the promotion of IVDD by CTGF, Liu et al^13^ elucidated that CTGF-transfected NP cells transplantation can reverse the percutaneous puncture induced rabbit IVDD. Additionally, Fujita et al^14^ reported VEGF-A contributes to the survival of NP cells during IVDD. However, the interaction underlying CTGF and VEGF in IVDD is not fully understood.

There are five members in the VEGF family, containing VEGF-A, placenta growth factor (PGF), VEGF-B, VEGF-C, and VEGF-D^15^, ^16^. VEGF-A is the earliest discovered subtype, and its primary role is to promote the formation of new blood vessels and increase vascular permeability. In this study, we deserved to explore the relation between CTGF and VEGF-A in the mediation of ECM synthesis of NP cells to discover the behavior of the interaction of these two in the progression of intervertebral disc fibrosis.

## Patients and methods

### Intervertebral disc sample

The research project was supported by the Ethics Committee of Affiliated Hospital of Jiangxi University of Traditional Chinese Medicine We obtained the patients' consent before the operations, which informed the specimens were taken for scientific research. Twenty-four lumbar disc specimens were obtained from orthopaedic surgery in our hospital, eight of which were donated from the patients who underwent spinal fractures without visible IVDD and required fixation and fusion operation. We selected the other sixteen patients undergoing lumbar disc herniation operations with disc degenerated grade 3 and 5, according to the Pfirrmann classification score^17^. We first conserved each specimen in a cell growth medium and brought them to the laboratory for the following experiments. This study was conducted in accordance with the Declaration of Helsinki.

### Intervertebral disc sample treatment

We separated the NP tissue from the samples and grouped them for histological and cellular studies. For histological analysis, the NP tissues were fixed in 4 % formalin solution for 24 h. The samples were then dehydrated with different gradients of ethanol, cleared with xylene, and embedded in paraffin. The Paraffin specimens were sectioned in 5 µm-thick and used for Masson staining and immunohistochemistry (IHC).

For the cellular study in vitro, we isolated the NP cell form the tissue as followed describe, NP tissues were minced and digested with 0.15% type II collagenase (Invitrogen, Carlsbad, CA, USA, diluted in Dulbecco's modified eagle medium (DMEM) solution, Keygen, Nanjing, China) for 6 h at 37°C until fragment was invisible. We got the primary NP cells after centrifuging the solution. All the cells were cultured to the first generation for the following treatments: NP cells were treated with human IL-1β protein (10 ng/mL, MCE, Shanghai, China) for a ranged duration from 1 day to 7 days. We also used recombinant human CTGF protein (Active) (ab269222, Abcam, Cambridge, MA, USA) and VEGF-A protein (ab155740, Abcam, Cambridge, MA, USA) to trigger the upregulation of CTGF and VEGF-A in NP cells. For the suppression of CTGF and VEGF-A, NP cells were transfected with short interfering RNA (siRNA) targeting CTGF or treated with the specific inhibitor of VEGF-A named Bevacizumab (BEV, Selleck, Houston, TX, USA).

### Masson and IHC staining

Paraffin sections were dewaxed with xylene and hydrated with gradient alcohol for the staining. We tested the fibrosis degree of the NP tissue by Masson staining kit (KGMST-8004, Keygen, Nanjing, China) according to the manufacturer's instructions, which makes collagen blue and fiber red.

For IHC, the sections were treated with 5 % goat serum solution for 10 min at room temperature and incubated with primary antibody (1: 100, rabbit polyclonal to VEGFA, ab9570, Abcam, Cambridge, MA, USA; 1:200, rabbit polyclonal to CTGF, ab6992, Abcam, Cambridge, MA, USA) at 4°C overnight, secondary antibody (1: 300 biotinylated goat anti-rabbit IgG, Beyotime, Shanghai, China) and processed with reagent SABC at room temperature for 30 min. Finally, sections were exposed with diaminobenzidine (DAB) (Solarbio, Beijing, China), and brown-yellow granular substance in the NP cells was be deemed too positive.

### Quantitative reverse-transcription polymerase chain reaction (RT-PCR)

Total cellular RNA was extracted according to the TRIzol instructions (Invitrogen, Carlsbad, CA, USA), and the content and concentration of the total RNA extracted were calculated by spectrophotometry. 2 µg of total RNA was reverse transcribed into complementary deoxyribose nucleic acid (cDNA), and then RT-PCR experiment was performed using an appropriate amount of cDNA as a template. The PCR reaction conditions were 95°C for 10 min; 95°C for 30 s, 60°C for 30 s, and 72°C for 30 s, for a total of 40 cycles. The relative quantification of the target gene was calculated using glyceraldehyde 3-phosphate dehydrogenase (GAPDH) as an internal reference. Primers for PCR are listed in Table 1, referring to the Primerbank (https://pga.mgh.harvard.edu/primerbank/).

**Table 1:** Primer sequences for RT-PCR

Gene name	Forward (5′>3′)	Reverse (5′>3′)
Collagen I	GAGGGCCAAGACGAAGACATC	CAGATCACGTCATCGCACAAC
Collagen II	TGGACGATCAGGCGAAACC	GCTGCGGATGCTCTCAATCT
Collagen III	ATGTTGTGCAGTTTGCCCAC	TCGTCCGGGTCTACCTGATT
Aggrecan	GGTGAACCAGTTGTGTTGTC	CCGTCCTTTCCAGCAGTC
CTGF	GAGCGGAGAGTCCTTCCAGAG	GGCCAAATGTGTCTTCCAGTC
VEGF-A	CTTGCCTTGCTGCTCTACC	CACACAGGATGGCTTGAAG
GAPDH	ACAACTTTGGTATCGTGGAAGG	GCCATCACGCCACAGTTTC

RT-PCR, quantitative reverse-transcription polymerase chain reaction

### siRNA transfection

The siRNA transfection method was performed according to the Lipofectamine2000 (Invitrogen, Carlsbad, CA, USA) product manual. NP cells were seeded in a 24-well plate at a density of 5000 per well, and 2 µL of CTGF-siRNA designed by the EnePharma (Shang-hai China), 1 µL of Lipofectamine 2000, and 50 µL of Opti-MEM I was used to preparing CTGF-siRNA-transfection reagent mixture. The NP cells were incubated with the siRNA-transfection reagent mixture for 6 h, and then we changed the medium. The sequences of the siR-NA-CTGF are listed as Table 2.

**Table 2:** Primer sequences of the siRNA-null and –CTGF

Gene name	Forward (5′>3′)	Reverse (5′>3′)
null	UUCUCCGAACGUGUCACGUTT	ACGUGACACGUUCGGAGAATT
CTGF-1	CCAGACCCAACUAUGAUUATT	UAAUCAUAGUUGGGUCUGGTT
CTGF-2	CCAAGCCUAUCAAGUUUGATT	UCAAACUUGAUAGGCUUGGTT
CTGF-3	GCUAAAUUCUGUGGAGUAUTT	AUACUCCACAGAAUUUAGCTT

### Western blotting

Total protein was extracted from NP cells of each group by radioimmunoprecipitation assay (RIPA) lysate (Invitrogen, Carlsbad, CA, USA), and protein concentration was determined by bicinchoninic acid (BCA) (Beyotime, Shanghai, China) method. 30 µg protein from each group was taken for gel electrophoresis and transferred to polyvinylidene fluoride (PVDF) membrane (Roche, Basel, Switzerland). After being blocked with 5% skim milk for 2 h at room temperature, the membranes were incubated with anti-VEGFA (1:1000, ab9570, Abcam, Cambridge, MA, USA), anti-CTGF (1:1000, ab6992, Abcam, Cambridge, MA, USA), and anti-GAPDH (1:1000, ab181602, Abcam, Cambridge, MA, USA) at 4°C overnight. The secondary antibody was added for incubation for another 1 h, and the membranes were developed by electrochemiluminescence (ECL) chemiluminescence (Beyotime, Shanghai, China) method for 1 min and exposed to X-ray films in a dark room. The results are expressed as the ratio of each protein to GAPDH gray value.

### Immunofluorescence (IF)

Before staining, NP cells were fixed with 4% paraformaldehyde for 15 min, permeabilized with 0.2% Triton-X-100 (Solarbio, Beijing, China) for 15 min, and blocked with bovine serum albumin (BSA) for 30 min. After that, the cells were incubated with the collagen I (ab34710, Abcam, Cambridge, MA, USA) and collagen II antibody (ab34712, Abcam, Cambridge, MA, USA) overnight at 4°C. Finally, goat anti-rabbit Dylight 488 fluorescent antibody (Invitrogen) and 4′,6-diamidino-2-phenylindole (DAPI) were added to incubate for another 1 h.

### Statistical analysis

All the data were analysed by GraphPad Prism 6.0 software (La Jolla, CA, USA) and expressed as mean ± standard deviation (Mean ± SD). Comparison between multiple groups was done using One-way ANOVA test followed by Post Hoc Test (Least Significant Difference). P<0.05 was considered to be statistically significant.

## Results

### CTGF and VEGF-A highly increased in the late-term of IVDD

**Figure 1:**
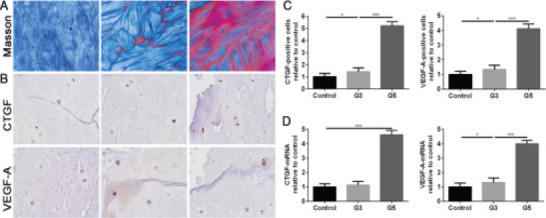
CTGF and VEGF-A highly expressed in the late-term of IVDD. NP tissues of different degeneration degrees were collected. (A) Representative images of Masson staining (magnification: 400×). (B) Representative images of IHC (magnification: 400×), and (C) quantification analysis. (D) mRNA level of CTGF and VEGF-A was determined by RT-PCR. The values are mean ± SD of three independent experiments. (*P<0.05, ***P<0.001)

The late phase of IVDD is severely fibrotic. Collagens I and III are expressed in large quantities. Additionally, collagen II and aggrecan are gradually degraded, resulting in the proportion of the original collagen gradually changes. To intuitively represent the fibrotic progression of the IVDD, the Masson staining was used to emerge the fibrosis in the NP tissue. As shown in Figure 1A, we selected three different conditions of NP tissue, containing the no degenerated disc from the lumbar vertebral fracture (control group), the degenerated grade 3 disc of Pfirrmann classification score (G3, middle-term degenerated group), and the degenerated grade 5 disc of Pfirrmann classification score (G5, late-term degenerated group). We did not notice any visible fibrotic changes in the control group, but the fibrotic area was observed in the G3 group, which was significantly marked in the G5 group (Figure 1A). To determine the CTGF and VEGF-A expression in the progression of IVDD, we used the IHC and RT-PCR to test this two-gene in both protein and mRNA levels. The result from the IHC suggested that the CTGF and VEGF-A positive cells were slightly increased in the G3 group compared to the control, but it was increased with roughly five or four-fold growth in the G5 group compared to the control (Figure 1B and 1C). Apart from this, the variation tendency of VEGF-A mRNA expression was similar to the protein level. Whereas, the increase of CTGF mRNA expression was only significant in the G5 group but not in the G3 phase (Figure 1D). The data indicated that the fibrosis was much severe in the late-term of IVDD, and the expression of CTGF and VEGF-A was not highly increased until the late phase of IVDD.

### CTGF and VEGF-A increased markedly in the late-term fibrosis of NP cells

**Figure 2:**
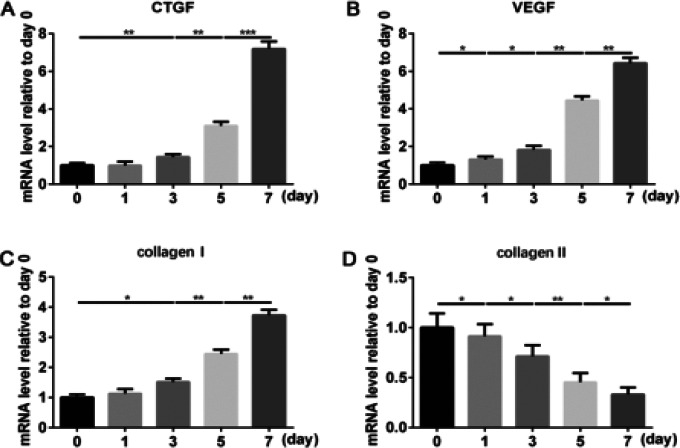
CTGF and VEGF-A highly expressed in the late-term of NP cell degeneration. NP cells were treated by IL-1β (10 ng/ml) from 1 day to 7 days. (A-D) The mRNA expression level of CTGF, VEGF-A, collagen I, and aggrecan II was assayed by RT-PCR. The values are mean ± SD of three independent experiments. (*P<0.05, **P<0.01, ***P<0.001)

To further explore the CTGF and VEGF-A expression in the degenerated NP in vitro, we isolated the NP cells and induced NP cells degeneration via the stimulation of IL-1β up to 7 days. We observed the dynamic changes of the mRNA expression of CTGF and VEGF-A at the time point of 1,3,5 and 7 days under the IL-1β stimulation. The result of the RT-PCR suggested that CTGF was increased after 3 days' treatment, which was much more evident with the increased treated time (Figure 2A). Though, we observed the upregulation of VEGF-A on the first day of stimulation, the increase of which was not much compared to day 0. However, the mRNA expression of VEGF-A was highly upregulated on day 5 and day 7 compared to day (Figure 2B). Additionally, the mRNA expression of collagen I and II was also analyzed to predict the fibrosis of NP cells. As expected, the result suggested the mRNA level of collagen I was gradually increased caused by IL-1β (Figure 2C); on the contrary, collagen II was progressively decreased in 7 days' stimulation (Figure 2D). Therefore, we concluded that the CTGF and VEGF-A were both increased in the fibrotic NP cells, which was much significant and marked in the late-term of degeneration in vitro.

### The interaction between CTGF and VEGF-A in NP cells in vitro

**Figure 3:**
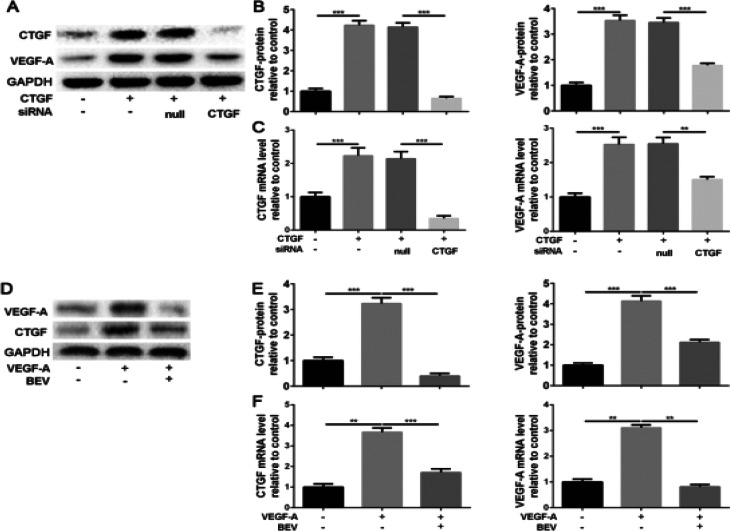
The interaction between CTGF and VEGF-A in NP cells in vitro. NP cells were treated with exogenic CTGF (2 ng/mL) or VEGF-A (3 ng/mL) protein for 3 days to upregulated the CTGF and VEGF-A expression of NP cells. Meanwhile, the siRNA transfected NP cells targeting null or CTGF were treated with exogenic CTGF protein (5 ng/mL) for 3 days as well. Besides, BEV (20 ng/mL) was also used to co-cultured NP cells with VEGF-A protein (3 ng/mL) for 3 days. (A, D) The protein expression level of CTGF and VEGF-A was determined by Western blotting and (B, E) quantification analysis. (C, F) The mRNA expression level of CTGF and VEGF-A was assayed by RT-PCR. The values are mean ± SD of three independent experiments. (**P<0.01, ***P<0.001)

To determine the interaction between CTGF and VEGF-A in the NP cells, we mediated them gene expression by exogenic protein stimuli, siRNA transfection, or inhibitor suppression to detect the effect on the other. We treated the NP cells with CTGF active protein for 3 days, and the CTGF was significantly increased compared to control. Simultaneously, the VEGF-A protein was upregulated as well. Apart from this, we used negatively controlled siR-NA (null-siRNA) transfected, and CTGF-siRNA transfected NP cells for CTGF protein treatment. Null-siRNA did not affect the CTGF expression of NP cells, but the siRNA targeting CTGF was efficient in suppressing the CTGF protein expression compared to the null-siRNA group, which also led to the reduction of VEGF-A protein expression (Figure 3A and 3B). Besides, the changes in mRNA expression caused by CTGF protein treatment and siRNA transfection presented a similar tendency as the protein expression (Figure 3C). The change of CTGF gene expression triggered the change of VEGF-A expression. We also wondered whether the CTGF expression would be positively related to VEGF-A distribution. Therefore, we used exogenic VEGF-A protein to upregulated the VEGF-A protein expression, which significantly resulted in the overexpression of CTGF protein. However, as well as VEGF-A, the protein expression of CTGF has decreased again due to the VEGF-A specific inhibitor BEV (Figure 3D and 3E). What's more, the tendency of mRNA expression was corresponding to the result of protein (Figure 3F). Summary, CTF can positively affect the CTGF-A expression, and CTGF-A can also positively affect the CTGF expression.

### The interaction between CTGF and VEGF-A in the fibrotic progression of NP cells

**Figure 4:**
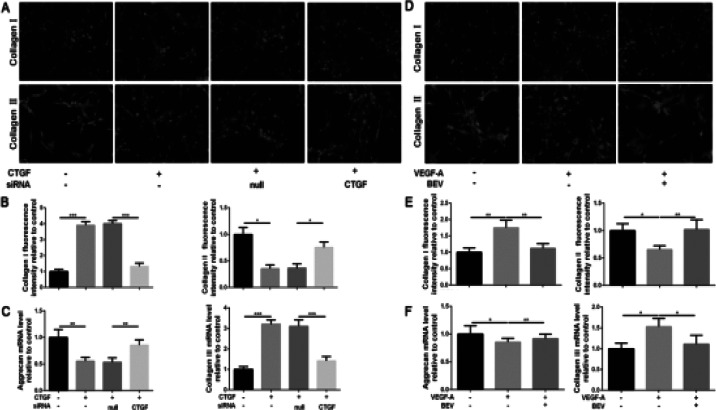
The upregulation of CTGF and VEGF-A accelerated the fibrotic progression of NP cells. NP cells were treated, as mentioned above. (A, D) IF staining of collagen I and II (magnification: 400×), and (B, E) quantification analysis. (C, F) The mRNA expression level of aggrecan and collagen III was assayed by RT-PCR. The values are mean ± SD of three independent experiments. (*P<0.05, **P<0.01, ***P<0.001)

CTGF and VEGF-A are responsible for the fibrosis of NP. Therefore, we finally analysed the fibrotic related gene expression, containing collagen I and collagen III, and collagen II and aggrecan the most critical ECM components synthesized by NP cells. We treated the NP cells with CTGF protein and transfected with siRNA, as mentioned above. As shown in Figure 4A-4C, the collagen I and collagen III expression were positively related to the expression of CTGF, and collagen II and aggrecan levels were negatively associated with the expression of CTGF. Similar to CTGF, VEGF-A played a role in the mediation of collage distribution as well. Collagen I protein and collagen III mRNA expression were increased caused by VEGF-A stimulation and were decreased again at the presence of BEV stimulation. Besides, collagen II protein and aggrecan mRNA synthesis was affected by the upregulation of VEGF-A but recovered after BEV interruption (Figure 4D-4F). Therefore, the activation of CTGF or VEGF-A accelerates the fibrosis and ECM destruction of NP cells, and the suppression of CTGF or VEGF-A could alleviate the fibrosis and ECM destruction of NP cells.

## Discussion

IVDD mainly occurs in the NP of the inner tissue of the disc, which is composed of NP cells and ECM. The former is derived from MSCs and has the role of maintaining the healthy metabolism of the NP1. However, the activity of NP cells is also affected by the physical structure and material components of the ECM that is mainly composed of water molecules, collagen, and aggrecan. In normal intervertebral discs, collagen I is concentrated in the outer layer of the annulus fibrosus (AF), and collagen III is mainly distributed around the annulus fibrosus cells. With the degeneration of the intervertebral disc, the component of collagen changes^18^. The amount of collagen I and III is further increased, and it replaces collagen II, which reduces the pressure and deformation of the NP, and eventually, the entire disc becomes fibrotic^19^. The increase of CTGF concentration and the unregulated autocrine regulation mechanism led to the redistribution of collagen expression. CTGF can repair the NP in the early stage of degeneration^9^, ^20^. However, CTGF is closely related to the fibrosis process in the late stage of IVDD^5^. In our experiment, CTGF was mildly increased in the early stage of IVDD but highly increased in the severely degenerated disc. Hence, we hold the opinion that CTGF has a dual effect during the development of IVDD, it can regulate ECM, promote collagen II and aggrecan synthesis, and slow down the degeneration process in the early stage. In the middle and late stages, due to cell degeneration, necrosis, and apoptosis, the number of NP cells gradually decreases, and cell function is also significantly reduced. Coupled with the accelerated degradation of collagen II and aggrecan, CTGF stimulates to produce collagen I and III at the transcription levels; thereby, interstitial fibers continue to proliferate, accelerating the process of disc degeneration.

CTGF plays an essential role in human tissue and organ fibrosis. It is a critical factor in the fibrotic cytokine network, which is closely related to the occurrence and development of fibrotic diseases^21^, ^22^. During the process, there may be new blood vessels, and CTGF promotes the adhesion and proliferation and migration of vascular endothelial cells and induces the formation of capillaries^23^. Ali et al^10^ found that the expression of CTGF in the angiogenesis area of the intervertebral disc is higher than that of the healthy disc, which proves that CTGF promotes disc degeneration relating to angiogenesis. Peng et a^15^ reported that fibrotic NP has prominent neovascularization and significantly increased CTGF expression, which may be one of the causes of low back pain. It is believed that CTGF may be a joint molecule that studies the mechanism of angiogenesis to promote the degeneration of intervertebral discs, which is the key question we want to elucidate in this study.

The expression of VEGF is different in the intervertebral discs at different periods. During fetal development and maturation of the intervertebral discs require a lot of nutrition. These requirements are met by the blood supply from the outer layer of the annulus fibrosus (AF)^24^. VEGF plays a vital role in the formation and maintenance of blood vessels in the AF. The nutrients from the endplate and the AF are sufficient to maintain disc metabolism and function, VEGF expression decreases, and the disc gradually degenerates into avascular tissue. With the aging of the intervertebral disc gradually degenerate, VEGF is again highly expressed at this time, which may be a manifestation of the body's attempt to repair damage and delay degeneration^25^. In particular, vascularization may increase the blood supply of the AF, provide nutrition for degenerative tissues, and slow the degeneration of the NP^26^. But with the prolongation of the IVDD, the new blood vessels increase the infiltration of monocytes, proteolytic enzymes, and inflammatory reactions, which accelerated the degeneration and fibrosis of ECM. Therefore, the role of VEGF, which is similar to CTGF, is continuously changing during the progression of IVDD.

In consequence, we hypothesized there exists an interaction between the CTGF and VEGF in the IVDD. It has been mentioned about CTGF and angiogenesis promote each other in some literature. In human synovial fibroblasts, CTGF mediates an increase in VEGF expression via regulating miR-210 expression^27^. In bovine retinal endothelial cells, VEGF can induce the production of CTGF^28^. VEGF up-regulates the expression of CTGF protein by the activation of the Akt/PKB pathway and the mediation of NF-κB. From the data we collected, the increased CTGF significantly triggered the VEGF-A expression, so as to the fibrosis of NP, likewise, the upregulation of VEGF-A also caused CTGF expression and led to the NP fibrosis.

## Conclusions

In conclusion, our study elucidates that the interaction between CTGF and VEGF-A contributes to the fibrotic progression of IVDD. The novel findings can help understand the mechanism underlying the relation between CTGF and VEGF-A in the NP cells and apply a strategy to suppress intervertebral disc fibrosis by interrupting the gap between CTGF and VEGF-A.
